# Validity and Reliability of a Questionnaire Measuring Knowledge, Attitude, and Practice Regarding Dementia Among General Population and Healthcare Workers in Urban India

**DOI:** 10.7759/cureus.28196

**Published:** 2022-08-20

**Authors:** Zarrin Ansari, Akanksha Togra, Rajmohan Seetharaman, Abhilasha Rashmi, Sudhir Pawar, Manish Pawar

**Affiliations:** 1 Pharmacology and Therapeutics, Lokmanya Tilak Municipal Medical College and General Hospital, Mumbai, IND; 2 Biostatistics and Epidemiology, Lokmanya Tilak Municipal Medical College and General Hospital, Mumbai, IND

**Keywords:** knowledge level, perception, alzheimer’s disease, dementia, awareness

## Abstract

*Objective*: The present study aimed to validate a questionnaire and measure the previous knowledge, attitude, and practice (KAP) of the general population and healthcare professionals regarding the debilitating disorder of dementia.

*Design*: A questionnaire including 27 items was compiled by the authors and was circulated via the online platform.

*Setting*:A questionnaire-based survey was conducted using the online modality.

*Participants*: A convenience sampling method was used to recruit participants aged 18 and above from all walks of life.

*Measurements*: Test-retest reliability, item analysis, and Cronbach’s alpha were calculated for the compiled questionnaire. The responses of the participants were assessed using descriptive statistics and the chi-square test.

*Results*: A total of 503 responses were collected. The internal consistency (Cronbach's alpha=0.70) was acceptable and the test-retest reliability (0.823) was good. Eighty-one percent (408/503) of participants had heard the word dementia. Seven percent (27/408) of the participants who had heard the word dementia did not have any knowledge about the symptoms of dementia. Thirty-three percent (136/408) of participants believed that dementia could not be prevented. Almost half, i.e., 46% (187/408) of participants, considered dementia as a normal part of aging.

*Conclusions*: The present study provides a fully validated questionnaire, which could prove helpful in research as it permits generating high-quality data and reducing measurement error. Knowledge of dementia among the general participants seems to be moderate and prompts towards the development of advocacy programs.

## Introduction

According to the World Health Organization (WHO), around 50 million people have dementia worldwide as of September 2020, and there are nearly 10 million new cases every year [1]. The global population of people living with dementia has more than doubled from 1990 to 2016, mainly due to population aging and growth [2]. The Delphi Consensus states that the number of people affected with dementia will double every 20 years to 81.1 million by 2040. Most people with dementia have been reported to live in developing countries (60% in 2001, rising to 71% by 2040) [3]. The percentage prevalence reported in the year 2012 in India varies widely from 0.8 to 4.1 because of its multiethnicity, multicultural and environmental differences [4].

Awareness about dementia is vital towards its recognition and, thereby, its management. Unfortunately, awareness about it is lacking in various aspects, especially in developing countries. A study conducted in India demonstrated that the symptoms of cognitive impairment, especially in the early stages, are neglected by family caregivers. The symptoms are considered a normal part of aging [5].

The perception that dementia is a normal part of aging and cannot be prevented needs to be challenged. Prevention of dementia involves targeting modifiable factors, viz., unhealthy diet and lifestyle, hypertension, hypercholesterolemia, diabetes, and cardiovascular disease. A study from the Netherlands demonstrated that most people still are unaware of the association between lifestyle and brain health, indicating the need for public health awareness campaigns [6].

Dementia literacy, conceptualized as knowledge and beliefs regarding dementia that aid recognition, management, or prevention, should be a target for optimizing care and improving the quality of life of people [7]. The dementia burden encompasses national, familial, and individualistic involvements. Raising awareness is vital towards challenging the misconceptions about the disorder. A more informed public is more likely to approach the healthcare and other available resources to battle the long-term healthcare, legal, and social implications of dementia.

Objective understanding of dementia is scarce, especially among the general population of India. Only one knowledge, attitude, and practice (KAP) study could be found in the current literature which was conducted among the general population. One KAP study was conducted among the caregivers of patients with dementia and another among medical undergraduate students. An interesting study was conducted among Indians residing in the United Kingdom (UK) [8-11]. The present KAP study aims to bridge the gap in an objective understanding of dementia among the general population and current healthcare providers. The primary objective is to obtain information on various aspects of awareness and attitude on a public platform so that the information can influence future healthcare strategies and policies. A comparison of KAP has also been conducted among the general population and healthcare providers. Therefore, the compilation of a questionnaire that is convenient to use and has adequate validity and reliability is recommended. The secondary objective was to develop and adopt a KAP questionnaire in the local setting and determine its validity and reliability among the general Indian population and healthcare providers.

## Materials and methods

Study design and participants

The current study was a cross-sectional, observational, and questionnaire-based KAP survey conducted among adult contacts (≥18 years). Approval from the Institutional Ethic Committee was obtained. 

Compilation of questionnaire

A questionnaire was compiled in the English language after incorporating items from a survey available online [12]. The language was kept simple to be understood even by the general population. The questionnaire compiled for the current study consisted of 27 questions divided into three groups; the first five questions on the demography of the participants, the following eight questions on assessing knowledge, and the last 14 questions on attitude and practice. 

Data collection

The questionnaire was circulated on the online platform. The link to the survey was circulated among adult contacts of the authors to gather their responses. The general population and healthcare professionals were approached for the survey. The convenience sampling method was used to obtain data in this survey. The first question in the survey was mandatory and was directed towards obtaining participant consent. While filling out this questionnaire, participants were instructed to answer all mandatory questions. When all of the questions were answered, the participants were notified of the completion on the same online platform. The survey length was five to seven minutes. Thirty participants were randomly selected from the response forms and were requested to fill out the form again after a gap of one to three months to check for the test-retest reliability parameter of the questionnaire. No compensation was provided to the participants in this survey. 

Validation of questionnaire

Once all the data collection were completed, the data were analyzed for appropriateness, validity, and reliability.

Test-retest reliability

The calculation of test-retest reliability included all data values, viz., knowledge, attitude, and practice from the questionnaire. Two sets of responses were elicited by the same set of thirty participants within 30 days. The statistical test used to assess the agreement between the pre- and post-groups was intraclass correlation coefficients (ICCs) for categorical Likert scales, including five-point, three-point, and dichotomous data. With respect to ICC, a two-way random effect model along with absolute agreement was used for the calculation. The reliability classification of ICC values was interpreted as: less than 0.5 is indicative of poor reliability, between 0.5 and 0.75 is indicative of moderate reliability, between 0.75 and 0.9 is indicative of good reliability, and greater than 0.90 is indicative of excellent reliability [13].

Item Analysis

This involves statistical analysis of the results of a test administered to identify which items can be retained and which need to be discarded [14].

Internal Consistency

The tendency towards consistency found in repeated measurements of the same phenomenon is reliability. Internal consistency refers to how all of the items on a scale measure the different aspects of the same attribute [15]. Cronbach’s alpha is a measure of internal consistency within a questionnaire, that is, how closely related a set of items are as a group. The interpretation was done on the bases of α value; “Excellent” (α ≥ 0.9), “Good” (0.9 > α ≥ 0.8), “Acceptable” (0.8 > α ≥ 0.7), “Questionable” (0.7 > α ≥ 0.6), “Poor” (0.6 > α ≥ 0.5), and “Unacceptable” (0.5 ≥ α) [16].

Statistical analysis

All analyses were conducted using IBM SPSS Statistics version 23 (IBM Corporation Business Analytics Software, California) and alpha levels of p < 0.05 were considered significant. All raw data were converted into categorical scales for appropriate statistical analysis. The Kolmogorov-Smirnov test was applied to test the normality of the distribution of the answers. Demographic data were analyzed using descriptive statistics. The chi-square test was used to test differences between proportions of respondents in different groups, viz., participants related to healthcare and the general population. The initial descriptive analysis was conducted on the total number of participants. For further analysis of knowledge, attitude, and practice, the analysis was conducted on the participants who had marked “yes” for the question “Have you heard the word dementia?”.

## Results

Demographic variables

Five hundred and three responses were collected. The demographic details of the participants are given in Table 1. The survey included an equivalent number of males and females. Almost 90% (465/503) of participants had been to college or higher grades. The survey enrolled 42% (213/503) of participants related to healthcare in some way and 58% (290/503) of the general population. The 213 participants who had claimed themselves to be related to healthcare had mentioned their occupations as doctors, medical students/interns, paramedical staff, research coordinators, biostatisticians, dentists, healthcare product managers, and pharmaceutical sales representatives.

**Table 1:** Demographic variables.

Have you heard the word “dementia”?	Yes	No	Total
	N (%)	N (%)	
Total	408 (81.11%)	95 (18.88%)	503
Age group			
18 to 24 years	103 (78.62%)	28 (21.38%)	131
25 to 28 years	102 (82.93%)	21 (17.07%)	123
29 to 41 years	105 (84.00%)	20 (16.00%)	125
42 to 79 years	98 (79.03%)	26 (20.97%)	124
Gender			
Male	189 (79.75%)	48 (20.25%)	237
Female	216 (82.44%)	46 (17.56%)	262
Others	3 (75.00%)	1 (25.00%)	4
Education level			
School or less	19 (50.00%)	19 (50.00%)	38
College or higher	389 (83.66%)	76 (16.34%)	465
Related to healthcare in someway			
Yes	206 (96.71%)	7 (3.29%)	213
No	202 (69.66%)	88 (30.34%)	290

Interpretation for the reliability and validity tests

Test-Retest Reliability

The ICC single measure represents a value of 0.048 (95% CI: 0.027, 0.091), implying there is not much effect between the individual response variables when tested for pre- and post-groups. The average score observed in this study was 0.823 (95% CI: 0.719, 0.902) and was indicative of good reliability as per the aforementioned scale (Table 2).

**Table 2:** Intraclass correlation coefficient. **p-value < 0.01. #The estimator is the same, whether the interaction effect is present or not. $This estimate is computed assuming the interaction effect is absent because it is not estimable otherwise.

Type of intraclass correlation coefficient	Intraclass correlation	95% Confidence Interval		F-test with true value 0			
		Lower bound	Upper bound	Value	df1	df2	Sig
Single measures	0.048^#^	0.027	0.091	5.635	29	2639	0.000**
Average measures	0.823^$^	0.719	0.902	5.635	29	2639	0.000**

Item Analysis

An item analysis test was performed for all the variables in the questionnaire. When an item analysis test was run on the data variables, Cronbach’s alpha value was observed as 0.68 for 49 items in the questionnaire. The “item-total statistics for mean scale if items were deleted” was observed as 49.4509 on average for all the variables combined. Similarly, “the scale variance if an item is deleted” was observed as 54.3046 on average. “The average corrected item-total correlation” and “the average Cronbach’s alpha value if an item is deleted” observed were 0.1588 and 0.6771, respectively. There wouldn’t be much deviation in Cronbach’s alpha value if items were deleted, implying that the tool is reliable.

Cronbach’s Alpha

The individual items in the questionnaire were of various categories, ranging from a few open-ended questions, continuous data, dichotomous data, and Likert’s five-point and three-point scale. The final value of Cronbach’s alpha for the whole questionnaire was observed as 0.70, and the internal validity of the questionnaire was regarded as acceptable.

Results on Knowledge/Awareness, Attitude, and Practice

Out of the total 503 participants, 408 (81%) had heard the word dementia and 95 (19%) had not heard the word dementia. Seventy-six out of 465 (16%) participants who had attended college and higher studies had not heard the word dementia. Seven out of 213 participants (3.29%) who had claimed to have been related to healthcare had not heard the word dementia, whereas 88/290 (30%) of participants who were not related to healthcare in any way had not heard the word dementia (Table 1).

**Figure 1:**
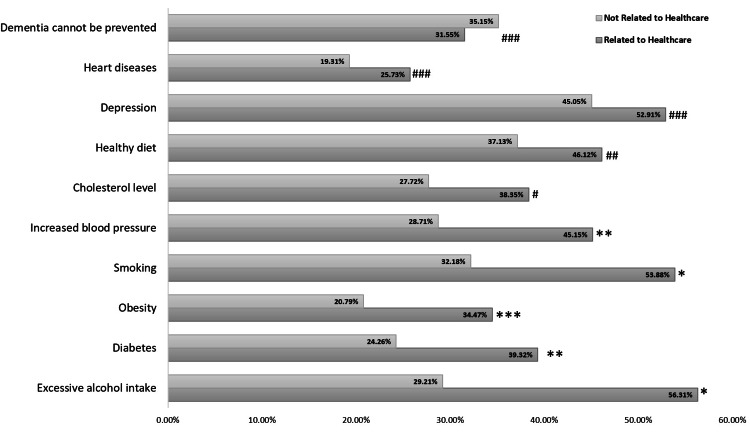
Percentage response regarding the modifiable risk factors for dementia. P-value: * = 0.000, ** = 0.001, *** = 0.002, # = 0.023, ## = 0.066, ### > 0.1.

Two hundred and six healthcare-related participants and 202 non-healthcare-related participants had heard the word dementia (Table 2). All participants who had not heard the word dementia were excluded from further analysis. Pertaining to the awareness regarding the prevention of dementia by modifying risk factors, 35% (71/202) of participants who were not related to healthcare and 32% (65/206) of participants who were related to healthcare believed that dementia could not be prevented. This difference was not statistically significant (p=0.441). Excessive alcohol intake, diabetes, obesity, smoking, increased blood pressure, and deranged cholesterol levels were considered crucial modifiable risk factors for preventing dementia by participants who were related to healthcare rather than the general population. The differences were statistically significant. Depression and an unhealthy diet were considered risk factors for dementia by 49% (200/408) and 42% (170/408) of participants, respectively (Figure 1).

**Figure 2:**
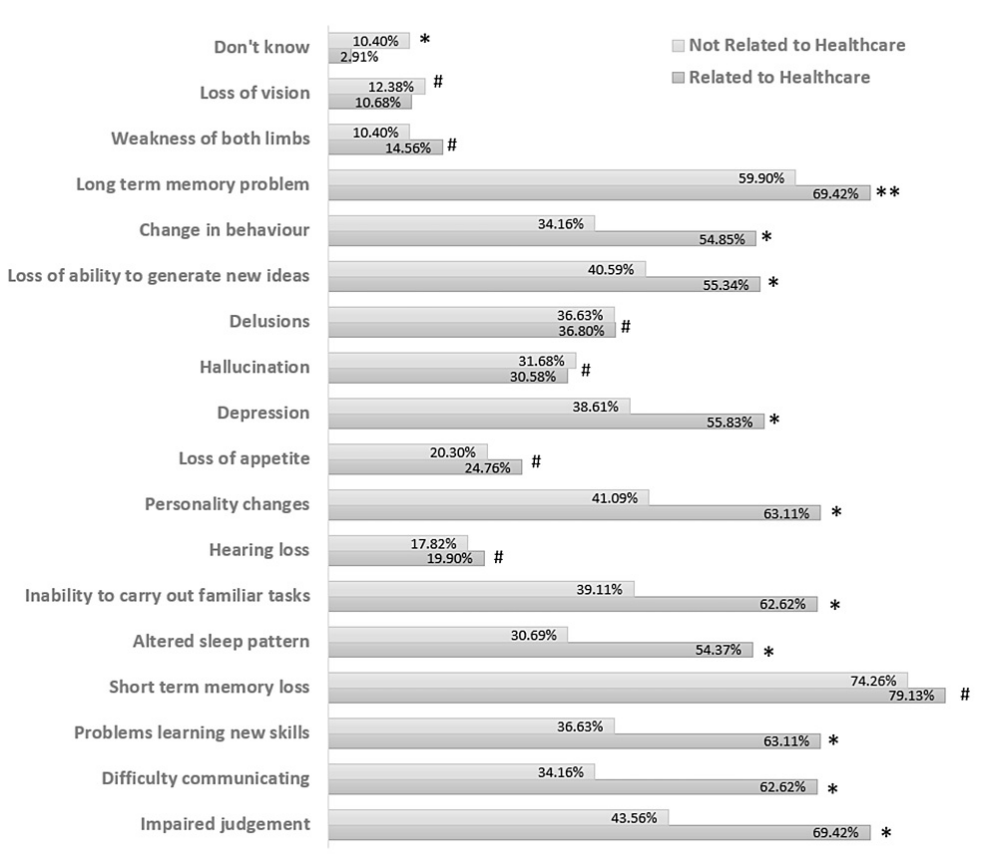
Percentage response regarding the symptoms experienced by patients of dementia. P-value: * < 0.005, ** = 0.044, # > 0.1.

Regarding the knowledge about the symptoms of dementia, 10% (21/202) of participants not related to healthcare and 3% (6/206) of participants related to healthcare marked the option “don’t know” in the survey (p=0.002). A total of 7% of participants marked the “don’t know” option in the survey. The highest chosen options were long-term (65%) and short-term memory loss (77%) by all participants. The lowest chosen options by all participants were hearing loss (19%), loss of appetite (23%), weakness of both limbs (13%), and loss of vision (12%). The options which had not been marked differently by the healthcare workers and the general population were; hearing loss (19.90% vs. 17.82%, p=0.591), loss of appetite (24.76% vs. 20.30%, p=0.281), hallucination (30.58% vs. 31.68%, p=0.81), delusions (36.89% vs. 36.63%, p= 0.957), weakness of both limbs (14.56% vs. 10.40%, p=0.203), loss of vision (10.68% vs. 12.38%, p=0.870), and short-term memory loss (79.13% vs. 74.26%, p=0.245) (Figure 2). 

Fifty-six percent (231/408) of participants identified the old age group as most affected by dementia. Almost 46% (187/408) of participants considered dementia is a normal part of aging. This question was answered affirmatively by 52% (106/206) of participants related to healthcare and 40% (81/202) of participants not related to healthcare. Forty-one percent (166/408) of participants considered dementia as hereditary. Forty-one percent (169/408) of participants believed that dementia could be cured with medication. Thirty-eight percent (78/206) of participants related to healthcare considered that dementia could be cured with medication (Table 3).

**Table 3:** Percentage responses regarding knowledge, attitude, and practice on dementia. *p-value > 0.05. **p-value > 0.01.

Are you related to healthcare in some way?	Yes n (%)	No n (%)	Total N (%)	p-value
Which age group does dementia affect?				
Adults > 18 years	5 (2.43)	5 (2.48)	10 (2.45)	0.003**
Old age group ≥ 65	134 (65.05)	97 (48.02)	231 (56.62)	
Any age	32 (15.53)	39 (19.31)	71 (17.40)	
Don't know	35 (17.99)	61 (30.20)	96 (23.53)	
Do you think dementia is a normal part of aging?				
Yes	106 (51.46)	81 (40.10)	187 (45.83)	0.000**
No	87 (42.23)	76 (37.62)	163 (39.95)	
Don't know	13 (6.31)	45 (22.28)	58 (14.22)	
Is dementia hereditary (family linked, i.e., if your parents have/had dementia, then you shall get it too)?				
Yes	101 (49.03)	65 (32.18)	166 (40.69)	0.000**
No	78 (37.86)	69 (34.16)	147 (36.03)	
Don't know	27 (13.11)	68 (33.66)	95 (23.28)	
Is dementia a healthcare priority?				
Yes	165 (80.10)	126 (62.38)	291 (71.32)	0.000**
No	27 (13.11)	20 (9.90)	47 (11.52)	
Don't know	14 (6.80)	56 (27.72)	70 (17.16)	
Do you think people with dementia can be cured with medication?				
Yes	78 (37.86)	91 (45.05)	169 (41.42)	0.000**
No	88 (42.72)	40 (19.80)	128 (31.37)	
Don't know	40(19.42)	71 (35.15)	111 (27.21)	
Do you know anyone, or have you known anyone with dementia?				
Yes	83 (40.29)	43 (21.29)	126 (30.88)	0.000**
No	123 (59.71)	159 (78.71)	282 (69.12)	
As you get older, do you worry about developing dementia?				
Yes	107 (51.94)	93 (46.04)	200 (49.02)	0.259
No	70 (33.98)	69 (34.16)	139 (34.07)	
Don't know	29 (14.08)	40 (19.80)	69 (16.91)	
As a carer or family member of the patient, have you hidden the diagnosis of dementia from people?				
Yes	10 (4.85)	20 (9.90)	30 (7.35)	0.005**
No	89 (43.20)	59 (29.21)	148 (36.27)	
Not applicable	107 (51.94)	123 (60.89)	230 (56.37)	
Has your health suffered as a result of the caring responsibilities of a relative with dementia?				
Yes	17 (8.25)	26 (12.87)	43 (10.54)	0.048*
No	73 (35.44)	51 (25.25)	124 (30.39)	
Not applicable	116 (56.31)	125 (61.88)	241 (59.07)	
Do you think doctors and nurses ignore people with dementia?				
Yes	44 (21.36)	24 (11.88)	68 (16.67)	0.000**
No	123 (59.71)	92 (45.54)	215 (52.70)	
Don't know	39 (18.93)	86 (42.57)	125 (30.64)	
Are you ashamed of having a relative with dementia?				
Yes	4 (1.94)	12 (5.94)	16 (3.92)	0.020*
No	119 (57.77)	94 (46.53)	213 (52.21)	
Not applicable	83 (40.29)	96 (47.52)	179 (43.87)	
You worry about close family members or close friends developing dementia.				
Yes	135 (65.53)	118 (58.42)	253 (62.01)	0.011**
No	47 (22.82)	38 (18.81)	85 (20.83)	
Don't know	24 (11.65)	46 (22.77)	70 (17.16)	
If you were experiencing memory problems, would you see a doctor?				
Yes	173 (83.98)	137 (67.82)	310 (75.98)	0.001**
No	17 (8.25)	29 (14.36)	46 (11.27)	
Don't know	16 (7.77)	36 (17.82)	52 (12.75)	
If someone close to you was experiencing a memory problem, would you encourage them to see a doctor?				
Yes	190 (92.23)	174 (86.14)	364 (89.22)	0.098
No	6 (2.91)	7 (3.47)	13 (3.19)	
Don't know	10 (4.85)	21 (10.40)	31 (7.60)	
If you were suffering from dementia, would you like to be told your diagnosis?				
Yes	192 (93.20)	168 (83.17)	360 (88.24)	0.003**
No	8 (3.88)	12 (5.94)	20 (4.90)	
Don't know	6 (2.91)	22 (10.89)	28 (6.86)	
If someone you cared for was suffering from dementia, would you want them to be told the diagnosis?				
Yes	173 (83.98)	154 (76.24)	327 (80.15)	0.131
No	19 (9.22)	25 (12.38)	44 (10.78)	
Don't know	14 (6.80)	23 (11.39)	37 (9.07)	
Is the diagnosis of dementia like a death sentence?				
Yes	21 (10.19)	25 (12.38)	46 (11.27)	0.000**
No	173 (83.98)	125 (61.88)	298 (73.04)	
Don't know	12 (5.83)	52 (25.74)	64 (15.69)	
Is it worse for family and friends than for the person with dementia?				
Yes	94 (45.63)	76 (37.62)	170 (41.67)	0.000**
No	75 (36.41)	49 (24.26)	124 (30.39)	
Don't know	37 (17.96)	77 (38.12)	114 (27.94)	
Do you think people with dementia should be allowed to drive?				
Yes	15 (7.28)	20 (9.90)	35 (8.58)	0.012**
No	168 (81.55)	140 (69.31)	308 (75.49)	
Don't know	23 (11.17)	42 (20.79)	65 (15.93)	

Only 31% (126/408) of participants had known anyone with dementia in their lifetime. Regarding the attitude and practice pertaining to dementia, almost half of the participants (200/408) were worried about developing dementia in old age. Among these 200, 107 participants related to healthcare, and the remaining 93 participants not related to healthcare, were worried about developing dementia in old age. Similarly, 62% (253/408) of participants were worried about their relatives or friends developing dementia in old age (Table 3).

Seven percent (30/408) of participants hid the diagnosis of dementia from people versus 36% (148/408) of participants who did not hide the diagnosis of dementia from people. On similar lines, only 4% (16/408) of participants were ashamed, and 52% (213/408) were not ashamed of having a relative with dementia. Eighty-eight percent (360/408) of participants expressed their desire to be told the diagnosis to themselves if they developed dementia. Almost 5% (20/408) of participants did not want to be told the diagnosis of dementia if they developed the disorder. More than 70% of participants mentioned encouraging their relatives to see a doctor if they developed memory problems (Table 3).

Only 11% (43/408) of participants considered that their health suffered due to the caring responsibilities of a relative with dementia versus 30% (124/408) of participants who disagreed with the same. On asking a similar question, almost 42% (170/408) of participants considered it worse for family and friends than for the person with dementia versus 30% (124/408) of participants who disagreed with the same (Table 3).

A whopping 73% (298/408) did not consider the diagnosis of dementia as a death sentence versus 11% (46/408) who did so. Almost 17% (68/408) believed that doctors and nurses ignored people with dementia versus 52% (215/408) who did not have the same opinion. Only 9% (35/408) of participants believed that people with dementia should be allowed to drive versus 75% (308/408) who did not consider the same (Table 3). 

## Discussion

Establishing a standardized instrument to measure knowledge about dementia is critical to assessing people's understanding of dementia and developing effective educational programs. This study provides preliminary evidence for the acceptability, reliability, and validity of the 27-item questionnaire. The average interval between the first and second survey administrations among randomly selected 30 participants was 30 days. The Pearson’s correlation coefficient of the two survey administrations was 0.823 and Cronbach’s alpha was 0.70. Thus, the reliability was considered good and the internal consistency was acceptable. On comparing the data about the Alzheimer’s disease knowledge scale (ADKS) validated by Carpenter et al. in 2009, the Pearson’s correlation coefficient and Cronbach’s alpha of ADKS (0.81 and 0.71, respectively) were equivalent to the evaluated parameters in the current study [17]. On item analysis, there was not much deviation in Cronbach’s alpha value if items were deleted, implying that the tool is reliable.

In the present study, 81% of the survey participants had heard the word dementia. Simply being familiar with the word dementia is equivalent to being dementia literate, which will be an inaccurate assumption. On further evaluation, 7% (27/408) of the participants who had heard the word dementia did not have any knowledge about the symptoms of dementia. The most popular dementia symptom, viz., memory loss, was acknowledged by 77% (313/408) of participants. Thirty-three percent (136/408) of participants believed that dementia could not be prevented. The present study was not equipped to check the knowledge of different types of dementia. Thus, a survey on the preventability of vascular dementias could not be assessed. The lowest marked options were hearing loss, loss of appetite, weakness of both limbs, and loss of vision. Almost 57% (231/408) correctly marked old age as the age group affected by dementia, whereas 24% (96/408) did not know the answer to this question. Thus, it can be inferred that approximately half of the 408 (81%) participants who had heard the word dementia had reasonably sufficient knowledge of dementia. This result was starkly different from the results of the study conducted by Purandare et al., who demonstrated that only 20% of Indians knew dementia as a disorder of the brain. This study was conducted among Indians residing in the United Kingdom and utilized the Dementia Knowledge Questionnaire (DKQ) [11]. Another study conducted by Low and Anstey in Australia demonstrated that 82% of randomly selected people could correctly identify dementia from the vignette provided [7].

Whether dementia is a normal part of aging or not is a crucial question. It reflects not only the knowledge but also people’s attitudes towards mental health in the geriatric population. In the current study, almost half (46%, 187/408) of the participants considered dementia a normal part of aging, and 14% (58/408) did not know the answer to this question. Breining et al. reported that 74% of 2000 participants thought it was normal to lose a memory with aging [18]. Two-thirds of 859 participants in a Turkish study conducted by Sahin et al. considered dementia to be a normal occurrence among older people [19].

Surprisingly, 3% (7/213) of healthcare-related participants in the current study had never heard the word dementia. These participants came from allied scientific backgrounds and none of them were medical personnel. A significantly higher percentage of participants not related to healthcare were not aware of any symptoms of dementia compared to healthcare professionals (10% vs. 3%, p=0.002). A significantly higher percentage of healthcare-related participants correctly marked the widespread symptoms of dementia, viz., impaired judgment, difficulty communicating, inability to learn new skills, long-term memory loss, altered sleep pattern, inability to carry out familiar tasks, personality changes, depression, loss of ability to generate new ideas and change in behavior as compared to the non-healthcare-related participants. The knowledge about other symptoms, viz., hearing loss, short-term memory loss, loss of appetite, hallucinations, delusions, weakness of limbs, and loss of vision, was not significantly different among the healthcare and non-healthcare-related participants.

It is evident from research that anti-dementia medications have a modest beneficial impact on neuropsychiatric and functional outcomes for patients with dementia [20]. In the current study, 41% (169/408) of participants believed that dementia could be cured with medications. Out of 169 participants who believed so, 46% (78/169) claimed that they were related to healthcare in some way. This was similar to the study by Siddiqui et al. from Pakistan, who reported that 41% of the participants viewed dementia as a curable condition [21]. These numbers reflect ignorance related to dementia, not only in the general population but also among participants related to healthcare.

Lopez et al. used qualitative methods to explore how stigma manifests within families from the perspective of caregivers of people with dementia. It was found that shame was a central theme because of which stigma was enacted and perpetuated. To manage the stigma, three types of behavior were evident, viz., silencing and not calling attention to the symptoms, concealing the diagnosis, and shunning and avoiding contact [22]. In the current study, 31% (126/408) of participants knew or had known someone with dementia and only 7% (30/408) of them revealed that they had hidden the diagnosis of dementia as a carer or family member of the patient. Almost 4% (16/408) revealed that they were ashamed of a relative with dementia. Though this questionnaire was not quantitatively equipped to measure shame among the caregivers, this data can help us understand that it is reasonably low among Indian caregivers despite a substantial level of ignorance about it.

Half of the participants in the current study reported that they worried about developing dementia as they grew older. Participants related to healthcare and the general population were equally worried about this parameter. Sixty-two percent of participants worried that their close family members or friends would develop dementia with time. In a study conducted by Werner in Israel, nearly 50% of the participants reported being very concerned about developing dementia [23]. In the present study, 76% of participants intended to seek medical help if they experienced memory problems. Almost 90% of participants intended to encourage relatives and friends to seek medical help if they experienced memory problems. More than 80% of participants expressed a desire to be informed about the diagnosis if they or their close ones develop dementia. Considering these results, it can be inferred that the general population was considerably worried about mental health in old age. However, a considerable portion of participants was open to seeking medical help for themselves and their near ones. The open attitude towards receiving the diagnosis and help is a major advantage towards improved condition management. A Dutch study by Commissaris et al. in the year 1993 reported that of all respondents who worried about their memory, 26% consulted their general practitioner to discuss their complaints [24]. This raises the possibility that over the 30 years, awareness and openness towards diagnosis and management of dementia might have improved. However, making crude inferences based on these data will be inappropriate as these studies were done in countries with different economic, social, demographic, and educational backgrounds. The social psychology of seeking formal medical care is based on motivations, beliefs, and perceptions regarding the condition [25].

The majority (76%) of participants believed that people with dementia should not be allowed to drive. Driving involves a complex set of cognitive actions affected in patients with dementia. The potential problems faced are forgetting how to locate familiar places, failing to observe traffic signs, making slow or poor decisions in traffic and driving at an inappropriate speed, among others. At some point in their lives, patients with dementia shall be unable to drive. Thus, planning is crucial towards making crucial driving-related decisions. However, it must be appreciated that withdrawing driving privileges has significant repercussions on the patient’s autonomy, social life, ability to access daily necessities, health care, and to survive independently in the community, especially if the patient’s spouse is unable to drive or the patient lives alone [26].

Eleven percent (46/408) of participants considered the diagnosis of dementia like a death sentence, and more than 70% (298/408) of participants did not have the same opinion. Dementia has an insidious onset, and the initial stages of mild cognitive impairment last for years [27]. Patients are independent for most of the early stages of the disorder as the majority of the cognitive functions are preserved [28]. The challenge here is the early diagnosis of this condition. Early diagnosis gives the patient and family a space to plan for the future, which can allay anxiety and depression of the deceased and carers to some extent [29].

Limitations

The present study has its limitations. The questionnaire was limited to only the English language. This was one of the important limitations. The language specification made it difficult to circulate the survey among people who did not know English or were uneducated. The online modality of delivering this survey limited access only to those participants who were familiar with technology and the internet. The survey encompassed only metropolitan cities, and thus the results do not reflect the mindsets of rural India. Half the participants were healthcare professionals, and thus, these survey results are not representative of the general population. The current study evaluates single and average internal validity. The results demonstrate that the single item internal validity is weak and the average internal validity/Cronbach’s alpha is standard. External validity has not been performed. Thus, it can be suggested that this tool may lead to measurement bias.

## Conclusions

The questionnaire assessed in this study proved to be a valid and reliable tool to measure knowledge of dementia. Since it is easy to understand and can be completed by the participants in a short time, it can be used in determining the knowledge level of the general public more readily and consistently. Implementing the questionnaire could identify areas where people are the most deficient. The present KAP study reflects that a considerable size of the general population as well as people related to healthcare foster ignorance about dementia. This estimate gives much room for constructing awareness programs to enlighten about this disabling disorder. The authors appreciate that despite a moderate awareness of dementia, the majority of the participants were not reluctant to embrace the diagnosis of dementia and approach medical help for the same. These findings do not reflect the pan-India population, and thus, similar studies need to be carried out on a larger scale to understand broader perspectives.

## Appendices

Questionnaire:

1) What are your initials?

2) What is your age in years?

3) What is your gender? Male/Female/Others

4) What is your education level? School or less/College or higher

5) Are you related to healthcare in someway? Yes/No

If Yes, mention your profession/designation

6) Have you heard the word “dementia”? Yes/No

7) Which age group does dementia affect?

·Children ≤ 12 years

·Teenagers 12-18 years

·Adults > 18 years

·Old age group ≥ 65 years

·Any age

·Don’t know

8) Do you think dementia is a normal part of aging? Yes/No/Don’t know

9) Is dementia hereditary (family linked, i.e., if your parents have/had dementia then you shall get it too) Yes/No/Don’t know

10) Is dementia a healthcare priority? Yes/No/Don’t know

11) Which of the following symptoms may a person with dementia experience? Tick as much as you think is correct.

·Impaired judgment

·Difficulty communicating

·Problems learning new skills

·Short-term memory loss

·Altered sleep pattern

·Inability to carry out familiar tasks

·Hearing loss

·Personality changes

·Loss of appetite

·Depression

·Hallucination (seeing or hearing things that are not present)

·Delusions (believing things that are not true)

·Loss of ability to generate new ideas

·Change in behavior (aggression and restlessness)

·Long-term memory problem

·Weakness of both limbs

·Loss of vision

·Don’t know

12) Do you think dementia can be prevented by modifying the factors given below? (Click those that you think to apply)

·Excessive alcohol intake

·Diabetes

·Obesity

·Smoking

·Increased blood pressure

·Cholesterol levels

·Healthy diet

·Depression

·Heart diseases

·Dementia cannot be prevented

13) Do you think people with dementia can be cured with medication? Yes/No/Don’t know

14) Do you know anyone or have you known anyone with dementia? Yes/No

If yes, please indicate their relationship to you.

15) As you get older do you worry about developing dementia? Yes/No/Don’t know

16) As a carer or family member of the patient, have you hidden the diagnosis of dementia from people? Yes/No/Not applicable

17) Has your health suffered as a result of the caring responsibilities of a relative with dementia? Yes/No/Not applicable

18) Do you think doctors and nurses ignore people with dementia? Yes/No/Don’t know

19) Are you ashamed of having a relative with dementia? Yes/No/Not applicable

20) You worry about close family members or close friends developing dementia.

Yes/No/Don’t know

21) If you were experiencing memory problems, would you see a doctor? Yes/No/Don’t know

22) If someone close to you was experiencing a memory problem, would you encourage them to see a doctor? Yes/No/Don’t know

23) If you were suffering from dementia, you would like to be told your diagnosis?

Yes/No/Don’t know

24) If someone you were caring for was suffering from dementia, would you want them to be told the diagnosis? Yes/No/Don’t know

25) Is the diagnosis of dementia like a death sentence? Yes/No/Don’t know

26) Is it worse for family and friends than for the person with dementia? Yes/No/Don’t know

27) Do you think people with dementia should be allowed to drive? Yes/No/Don’t know
